# HMG-CoA reductase is a potential therapeutic target for migraine: a mendelian randomization study

**DOI:** 10.1038/s41598-024-61628-9

**Published:** 2024-05-27

**Authors:** Kang Qu, Ming-xi Li, Peng Yu, International Headache Genetics Consortium, Aarno Palotie, Alice Pressman, Andrea C. Belin, Anna Bjornsdottir, Arn M. J. M. van den Maagdenberg, Aster V. E. Harder, Bendik S. Winsvold, Bertram Müller-Myhsok, Bru Cormand, Caroline Ran, Carrie Northover, Christian Kubisch, Cornelia van Duijn, Dale R. Nyholt, Daniel I. Chasman, Danielle Posthuma, Davor Lessel, Dorret I. Boomsma, Eija Hämäläinen, Espen S. Kristoffersen, Ester Cuenca-Leon, George Davey-Smith, Gisela M. Terwindt, Gudrun R. Sigurdardottir, Gyda Bjornsdottir, Heidi Hautakangas, Hreinn Stefansson, Irene de Boer, Jaakko Kaprio, Jes Olesen, John-Anker Zwart, Kari Stefansson, Lannie Ligthart, Lenore Launer, Linda M. Pedersen, Lisette J. A. Kogelman, Lyn R. Griffiths, M. Arfan Ikram, Maija Wessman, Mari Kaunisto, Maria G. Hrafnsdottir, Marjo Hiekkala, Marjo-Riitta Järvelin, Martin Dichgans, Matti Pirinen, Mikko Kallela, Mitja Kurki, Mona A. Chalmer, Nancy Pedersen, Olafur A. Sveinsson, Olli Raitakari, Padhraig Gormley, Patricia Pozo-Rosich, Priit Palta, Rainer Malik, Risto Kajanne, Sigrid Børte, Sigurdur H. Magnusson, Terho Lehtimäki, Thomas F. Hansen, Thorgeir E. Thorgeirsson, Tobias Freilinger, Tobias Kurth, Tonu Esko, Verneri Anttila, Ville Artto, Bai-hua Wu, Miao Shi, Ming Dong

**Affiliations:** 1 Department of Neurology and Neuroscience Center, The First Hospital of Jilin University, Xinmin Street #1, Changchun, 130021 China; 2 Department of Ophthalmology, The Second Hospital of Jilin University, Changchun, China; 3 Institute for Molecular Medicine Finland, Helsinki Institute of Life Science, University of Helsinki, Helsinki, Finland; 4 University of Helsinki, Helsinki, Finland; 5 Sutter Health, Sacramento, CA USA; 6 Department of Neuroscience, Karolinska Institutet, Stockholm, Sweden; 7 Neurology Private Practice, Laeknasetrid, Reykjavík, Iceland; 8 Department of Neurology, Leiden University Medical Centre, Leiden, The Netherlands; 9 Department of Human Genetics, Leiden University Medical Centre, Leiden, The Netherlands; 10 Department of Research, Innovation and Education, Division of Clinical Neuroscience, Oslo University Hospital, Oslo, Norway; 11 K.G. Jebsen Center for Genetic Epidemiology, Department of Public Health and Nursing, Faculty of Medicine and Health Sciences, Norwegian University of Science and Technology, Trondheim, Norway; 12 Department of Neurology, Oslo University Hospital, Oslo, Norway; 13 Max Planck Institute of Psychiatry, Munich, Germany; 14 Department of Genetics, Spain Centre for Biomedical Network Research on Rare Diseases, University of Barcelona, Barcelona, Spain; 15 23&Me Inc., Mountain View, CA USA; 16 Institute of Human Genetics, University Medical Center Hamburg-Eppendorf, Hamburg, Germany; 17 Department of Epidemiology, Erasmus University Medical Centre, Rotterdam, The Netherlands; 18 School of Biomedical Sciences, Faculty of Health, Centre for Genomics and Personalised Health, Centre for Data Science, Queensland University of Technology, Brisbane, QLD Australia; 19 Department of Medicine, Division of Preventive Medicine, Brigham and Women’s Hospital, Boston, MA USA; 20 Harvard Medical School, Boston, MA USA; 21 Department of Complex Trait Genetics, Center for Neurogenomics and Cognitive Research, Neuroscience Campus Amsterdam, VU University, Amsterdam, The Netherlands; 22 Netherlands Twin Register, Department of Biological Psychology, Vrije Universiteit, Amsterdam, The Netherlands; 23 Research and Communication Unit for Musculoskeletal Health, Department of Research, Innovation and Education, Division of Clinical Neuroscience, Akershus University Hospital and University of Oslo, Oslo, Norway; 24 Department of General Practice, Institute of Health and Society, University of Oslo, Oslo, Norway; 25 Department of Neurology, Akershus University Hospital, Lørenskog, Norway; 26 Pediatric Neurology Research Group, Vall d’Hebron Research Institute, Barcelona, Spain; 27 University of Bristol/Medical Research Council Integrative Epidemiology Unit, University of Bristol, Bristol, UK; 28 deCODE Genetics/Amgen Inc., Reykjavík, Iceland; 29 Danish Headache Center, Department of Neurology, Copenhagen University Hospital, Copenhagen, Denmark; 30 Institute of Clinical Medicine, Faculty of Medicine, University of Oslo, Oslo, Norway; 31 Laboratory of Epidemiology and Population Sciences, Intramural Research Program, National Institute on Aging, Bethesda, MD USA; 32 Centre for Genomics and Personalised Health, Queensland University of Technology, Brisbane, QLD Australia; 33 Department of Epidemiology, Erasmus University Medical Center, Rotterdam, The Netherlands; 34 Folkhälsan Research Center, Helsinki, Finland; 35 Landspitali University Hospital, Reykjavík, Iceland; 36 Department of Epidemiology and Biostatistics, MRC-PHE Centre for Environment and Health, School of Public Health, Imperial College London, London, UK; 37 Center for Life Course Health Research, Faculty of Medicine, University of Oulu, Oulu, Finland; 38 Unit of Primary Health Care, Oulu University Hospital, OYS, Oulu, Finland; 39 Department of Life Sciences, College of Health and Life Sciences, Brunel University London, London, UK; 40 Institute for Stroke and Dementia Research, University Hospital, LMU Munich, Munich, Germany; 41 Munich Cluster for Systems Neurology, Munich, Germany; 42 Department of Mathematics and Statistics, University of Helsinki, Helsinki, Finland; 43 Department of Public Health, University of Helsinki, Helsinki, Finland; 44 Department of Neurology, Helsinki University Central Hospital, Helsinki, Finland; 45 Psychiatric and Neurodevelopmental Genetics Unit, Department of Medicine, Massachusetts General Hospital, Boston, MA USA; 46 Department of Medical Epidemiology and Biostatistics, Karolinska Institutet, Stockholm, Sweden; 47 Centre for Population Health Research, University of Turku, Turku University Hospital, Turku, Finland; 48 Research Centre of Applied and Preventive Cardiovascular Medicine, University of Turku, Turku, Finland; 49 Department of Clinical Physiology and Nuclear Medicine, Turku University Hospital, Turku, Finland; 50 GSK Inc., Cambridge, MA USA; 51 Headache Unit, Neurology Department, Vall d’Hebron University Hospital, Barcelona, Spain; 52 Research and Communication Unit for Musculoskeletal Health, Department of Research, Innovation and Education, Division of Clinical Neuroscience, Oslo University Hospital, Oslo, Norway; 53 Department of Clinical Chemistry, Fimlab Laboratories, and Finnish Cardiovascular Research Center - Tampere, Faculty of Medicine and Health Technology, Tampere University, Tampere, Finland; 54 Novo Nordic Foundation Center for Protein Research, Copenhagen University, Copenhagen, Denmark; 55 Department of Neurology, Klinikum Passau, Passau, Germany; 56 Department of Neurology and Epileptology, Hertie Institute for Clinical Brain Research, University of Tuebingen, Tübingen, Germany; 57 Institute of Public Health, Charité – Universitätsmedizin, Berlin, Germany; 58 Estonian Biobank Registry, the Estonian Genome Center, University of Tartu, Tartu, Estonia; 59 Analytical and Translational Genetics Unit, Department of Medicine, Massachusetts General Hospital and Harvard Medical School, Boston, MA USA; 60 Program in Medical and Population Genetics, Broad Institute of MIT and Harvard, Cambridge, MA USA; 61 Stanley Center for Psychiatric Research, Broad Institute of MIT and Harvard, Cambridge, MA USA

**Keywords:** Statins, HMG-CoA reductase, Migraine, Mendelian randomization, Neuroscience, Neurogenesis

## Abstract

Statins are thought to have positive effects on migraine but existing data are inconclusive. We aimed to evaluate the causal effect of such drugs on migraines using Mendelian randomization. We used four types of genetic instruments as proxies for HMG-CoA reductase inhibition. We included the expression quantitative trait loci of the *HMG-CoA reductase* gene and genetic variation within or near the *HMG-CoA reductase* gene region. Variants were associated with low-density lipoprotein cholesterol, apolipoprotein B, and total cholesterol. Genome-wide association study summary data for the three lipids were obtained from the UK Biobank. Comparable data for migraine were obtained from the International Headache Genetic Consortium and the FinnGen Consortium. Inverse variance weighting method was used for the primary analysis. Additional analyses included pleiotropic robust methods, colocalization, and meta-analysis. Genetically determined high expression of HMG-CoA reductase was associated with an increased risk of migraines (OR = 1.55, 95% CI 1.30–1.84, *P* = 6.87 × 10^−7^). Similarly, three genetically determined HMG-CoA reductase-mediated lipids were associated with an increased risk of migraine. These conclusions were consistent across meta-analyses. We found no evidence of bias caused by pleiotropy or genetic confounding factors. These findings support the hypothesis that statins can be used to treat migraine.

## Introduction

Migraine, a common affliction endured by several people in headache clinics, is characterized by recurrent moderate-to-severe throbbing pain^1^. It affects ~ 12% of the global population and is the second most incapacitating ailment worldwide^2^. The deleterious impact of migraines on both well-being and quality of life cannot be underestimated because it imposes a substantial burden on a global scale^3^. Therefore, it is crucial to identify therapeutic targets for migraine to expand the available treatment options.

Observational studies have reported that disorders in the metabolism of lipids, including cholesterol and low-density lipoprotein cholesterol (LDL-C), may increase susceptibility to migraine^4,5^. Additionally, it has been speculated that certain lipid-lowering drugs such as statins possess migraine-ameliorating properties^6–8^. However, the available studies on the association between statins and migraine risk disagree, and the causal relationship remains uncertain^6–10^. Statins are a class of drugs that inhibit 3-hydroxy-3-methylglutaryl coenzyme A reductase (HMGCR). They have emerged as potent agents for reducing plasma LDL-C levels and play a crucial role in treating atherosclerotic diseases^11^. In addition to their lipid-lowering effects, statins can bolster the stability of atherosclerotic plaques, improve vascular endothelial function, mitigate oxidative stress and inflammation, and regulate autonomic nervous system function^12–14^. The efficacy and safety of statins in preventing ischemic stroke are firmly established, and they are widely used in clinical practice^11^. Accumulating evidence shows that migraine increases the risk of ischemic stroke^15,16^. Therefore, the discovery of the causal pathways of migraine related to lipids may contribute to improve our understanding of the mechanisms leading to the development of migraine. And it could also help us understand the relationship between migraine and stroke. Importantly, it may benefit the development of personalized treatment strategies, notably for individuals at a high risk of dyslipidemia or with a familial history of stroke.

Observational studies cannot eliminate confounding biases between exposure and outcomes. To circumvent this hurdle, Mendelian randomization (MR) analysis uses genetic variants (single nucleotide polymorphisms, SNPs) as instrumental variables to clarify the potential causal relationships between exposures and outcomes^17^. MR is comparable to randomized controlled trials in that genetic variations are randomly assigned at conception; therefore, MR can minimize interference by confounding bias^18^. Interference from reverse causality can also be avoided because genetic variants precede disease onset and are not affected by disease progression^18^. The expression of protein drug targets may be influenced by variants near the genes that encode them and such variants can therefore be used to predict potential clinical effects^19^. Drug-target MR uses genetic variants of genes encoding proteins of interest, usually cis-acting quantitative trait loci (cis-QTL), as instrumental variables to clarify the impact of the encoded proteins on disease outcomes^20^. When the protein of interest is likely to be the target of a drug’s action, MR analysis is referred to as drug-target MR^20^. If a drug is supported by genetic evidence, it indicates that the medication’s therapeutic impact and mechanism of action are supported by reliable scientific data and that the drug may be an effective treatment for the targeted the disease of interest.

In this study, we aimed to investigate the association between the risk of migraine and statin lipid-lowering agents using a two-sample drug-target MR design.

## Methods

We used publicly available genome-wide association studies (GWAS) and expression quantitative trait locus (eQTL) data. Informed consent and ethical approval had been obtained in all the original studies; therefore, no additional ethical approval was required for this study. Two-sample MR was performed according to the STROBE-MR guidelines^21^.

### MR assumptions and study design

**Figure 1:**
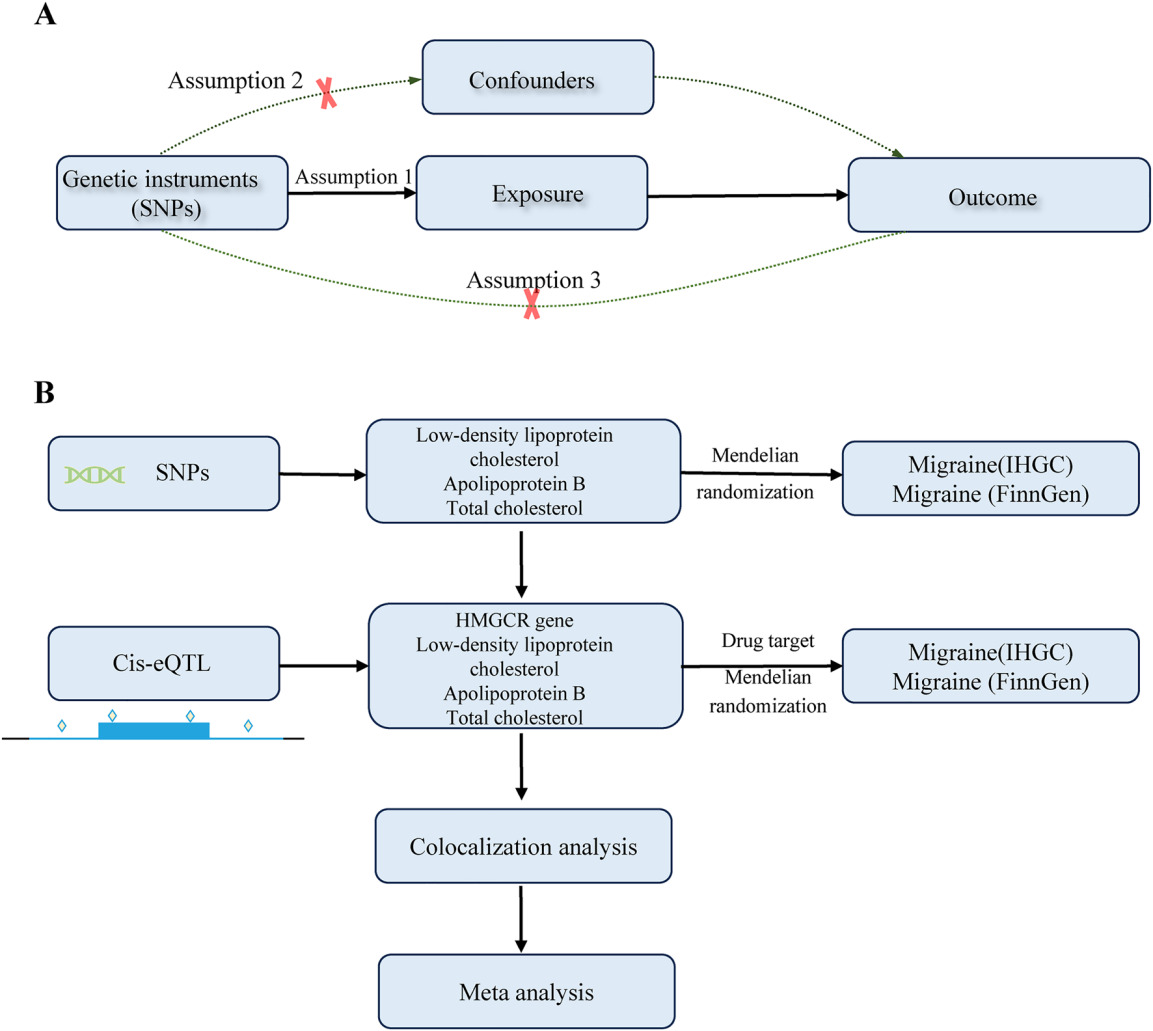
Overview of the study design in this study.

The selection of valid instrumental variables must satisfy three assumptions of MR analysis (Fig. 1)^17^. Given the observational association between circulating lipids and migraines, we first examined whether genetically predicted circulating lipids (LDL-C; apolipoprotein B, APOB; and total cholesterol, TC) were associated with migraines. Second, drug-targeted MR was performed to determine whether HMGCR expression in the blood affects migraines. Third, a colocalization analysis was performed to determine the presence of common genetic variants. Finally, to verify the observed associations, we assessed whether the levels of HMGCR-regulated LDL-C, APOB, and TC were associated with migraine. An overview of the study design is briefly outlined in Fig. 1.

### Genetic variation for exposure and identifying instrumental variables

Summary-level GWAS data for circulating lipids were obtained from the UK Biobank. The circulating lipids included LDL-C (n = 431,167)^22^, APOB (n = 439,214)^23^, and TC (n = 342,508)^24^. Age, sex, and type of genotyping chip were adjusted as covariates in the GWAS analyses of individuals of European ancestry included in the UK Biobank. The criteria for the selection of instrumental variables were as follows: ^1^ genome-wide significance (*P* < 5 e−08); (2) linkage disequilibrium (LD) at R^2^ < 0.001 within a 10,000-kb window based on the European-based 1000 Genomes Project; (3) palindromic SNPs and SNPs with minor allele frequencies < 0.01 removed; (4) proxies not sought for instrumental variables not available in the outcomes; and (5) F-statistics of instrumental variables calculated using the square of the standard error^25^, with an F-value > 10 suggesting sufficient instrument strength^26^.

The available eQTLs for HMGCR (n = 31,684) can serve as genetic proxies for statins, and summary data for eQTLs were obtained from the eQTLGen Consortium^27^. The instrumental variables used in this study were consistent with those reported by Huang et al.^28^. The following inclusions were used: ^1^ genome-wide significant association (*P* < 5 e−08); (2) defined as cis-eQTLs located within ± 100 kb windows around the coding gene; (3) a minor allele frequency > 1%; and (4) demonstrating independent association (low LD clumping r^2^ < 0.3).

Complementary analyses were conducted to verify the robustness of the associations obtained using cis-eQTLs as instrumental variables. Originally, we intended to use genetic instrumentation at the circulating HMGCR protein level (i.e., protein quantitative trait loci, pQTL) as a prerequisite for exposure validation. Unfortunately, we found no cis-acting pQTL that met these requirements. Therefore, considering that statins may affect the serum LDL-C, APOB, and TC levels, we selected these three circulating lipids as potential biomarkers^11,29,30^. We then selected genetic variants located within ± 100 kb of *HMGCR* (build GRCh37: chromosome 5:74,632,154–74,657,929) showing significant associations with LDL-C, APOB, and TC, respectively, at a genome-wide significance of *P* < 5 e−08, to serve as surrogates for statin therapy. LD was set to R^2^ < 0.1 within a 100-kb window, using a European reference panel from the 1000 Genomes Project. This method of selecting instrumental variables was also used in previous studies^28,31^. Triglycerides and high-density lipoprotein cholesterol were excluded because no instrumental variables that met the above criteria were extracted.

### Genetic variation for migraine

For the primary analysis, summary-level GWAS data for migraines were obtained from a meta-analysis conducted by the International Headache Genetics Consortium (IHGC), which included participants of European ancestry. The data were approved by a direct application and material transfer agreement^32^. The dataset used contained 48,975 migraine cases and 540,381 controls. Migraine cases were identified based on self-reported data or the International Classification of Headache Disorders. The cases included in this meta-analysis were adjusted for sex, age, and ancestry. The remaining details, including demographic characteristics, eligibility criteria, and ethical approval, can be found in the original article^32^. For replication analysis, summary statistics were obtained from the FinnGen study (nCase = 1,5905, nControl = 264,662, R8 release)^33^.

### Statistical analysis

#### MR analysis

The primary analytical method for MR is random-effects inverse variance-weighted (IVW), which assumes that all SNPs are valid instruments, allows for balanced pleiotropy, and provides the most precise estimates^34^. Additional sensitivity analyses included the MR–Egger intercept test^35^, the weighted median test^36^, the radial MR test^37^, and the MR pleiotropy residual sum and outliers test (MR-PRESSO)^38^. The MR–Egger detected horizontal pleiotropy, with *P* > 0.05 indicating none. The MR-PRESSO and radial MR methods were employed to identify outliers. And visualization methods such as scatter plots and leave-one-out plots are also used to identify outliers. Heterogeneity among the different IVs was evaluated using Cochran’s Q test. Burgess’s online calculator was used to calculate the power of the MR estimates^39^.

#### Bayesian colocalization analysis

To avoid the influence of LD or pleiotropy on MR findings, we performed a Bayesian colocalization analysis using the default parameters of the Coloc R package^40^. Bayesian colocation analysis was employed to assess the probability that the two traits (eQTL and migraine GWAS) shared the same causal variant^40,41^. We tested the posterior probabilities of five hypotheses: H0, not associated with any trait; H1/H2, associated with only one of the traits; H3, two traits having different causal variants; and H4, both traits having their causal SNPs and sharing the same SNP. We considered a posterior probability of hypothesis 4 (PPH4) > 0.8 (calculated by the Coloc.abf algorithm) as strong evidence for colocalization. For visualization, we used the “locuscomparer” R package^42^.

The Bonferroni method was employed to adjust the significance threshold for four exposures, requiring *P* < 1.25 × 10^−2^. Estimates were considered significant in the MR analysis when at least the IVW method estimates were significant and the three different MR methods were considered consistent in direction. The association results are presented as odds ratios (OR) with 95% confidence intervals (95% CI).

#### Meta-analysis

We performed a random-effects meta-analysis of the results obtained from the IHGC and FinnGen datasets to produce a comprehensive analysis of causality. The R package “Metafor” was used for this. The significance levels for the heterogeneity tests and the effect values of the meta-analysis results were set to 0.05.

#### Additional positive control analysis

Given the common and beneficial use of lipid-lowering medications in coronary artery disease, the statin-medicated condition was used as a positive control to evaluate the reliability of the instruments. A total of 122,733 patients and 424,528 controls were included in the GWAS data for coronary artery disease^43^.

R software (version 4.2.2; R Foundation for Statistical Computing, Vienna, Austria) was used for all statistical analyses^44^. The R package for MR analysis included “TwoSampleMR (version 0.5.6),” “MR-PRESSO (version 1.0),” “RadialMR (version 1.0),” “Coloc (version 1.0),” and “Metafor (version 1.0)”.

### Ethical approval and consent to participate

This study used data from published studies. All original studies have been approved by the corresponding ethical review board, and the participants have provided informed consent. In addition, no individual-level data was used in this study. Therefore, separate ethical approval was not required for this study.

## Results

Overall, 344 (LDL-C), 187 (APOB), and 259 (TC) SNPs were included in the analysis of the association between circulating lipids and migraine (Additional file 1: Supplementary Table 1). Two-sample MR analysis revealed no association between migraine and any of the three circulating lipids (Supplementary Tables 2–4). Seven eligible cis-eQTLs were included in the drug-targeted MRI analysis (Supplementary Table 5). From the GWAS summary-level data, 18, 10, and 11 SNPs within or near the *HMGCR* region were associated with LDL-C, APOB, and TC, respectively (Supplementary Table 5). The F-statistic of all included instrumental variables was greater than 10, indicating the absence of weak instrumental variable bias.

**Figure 2:**
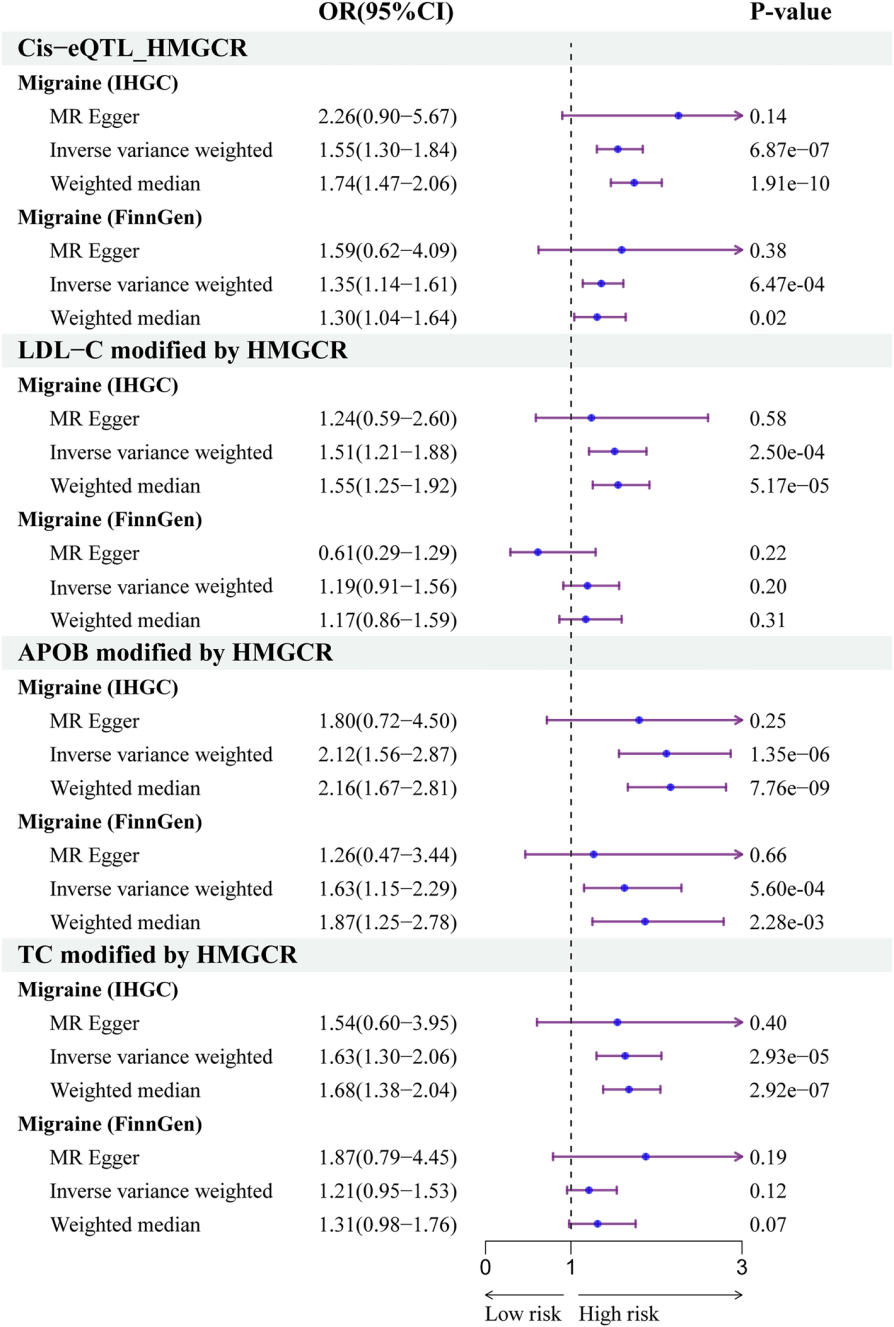
Associations linking HMG-CoA reductase gene expression or HMG-CoA reductase mediated low-density lipoprotein cholesterol, apolipoprotein B, and total cholesterol with the risk of migraine.

**Figure 3:**
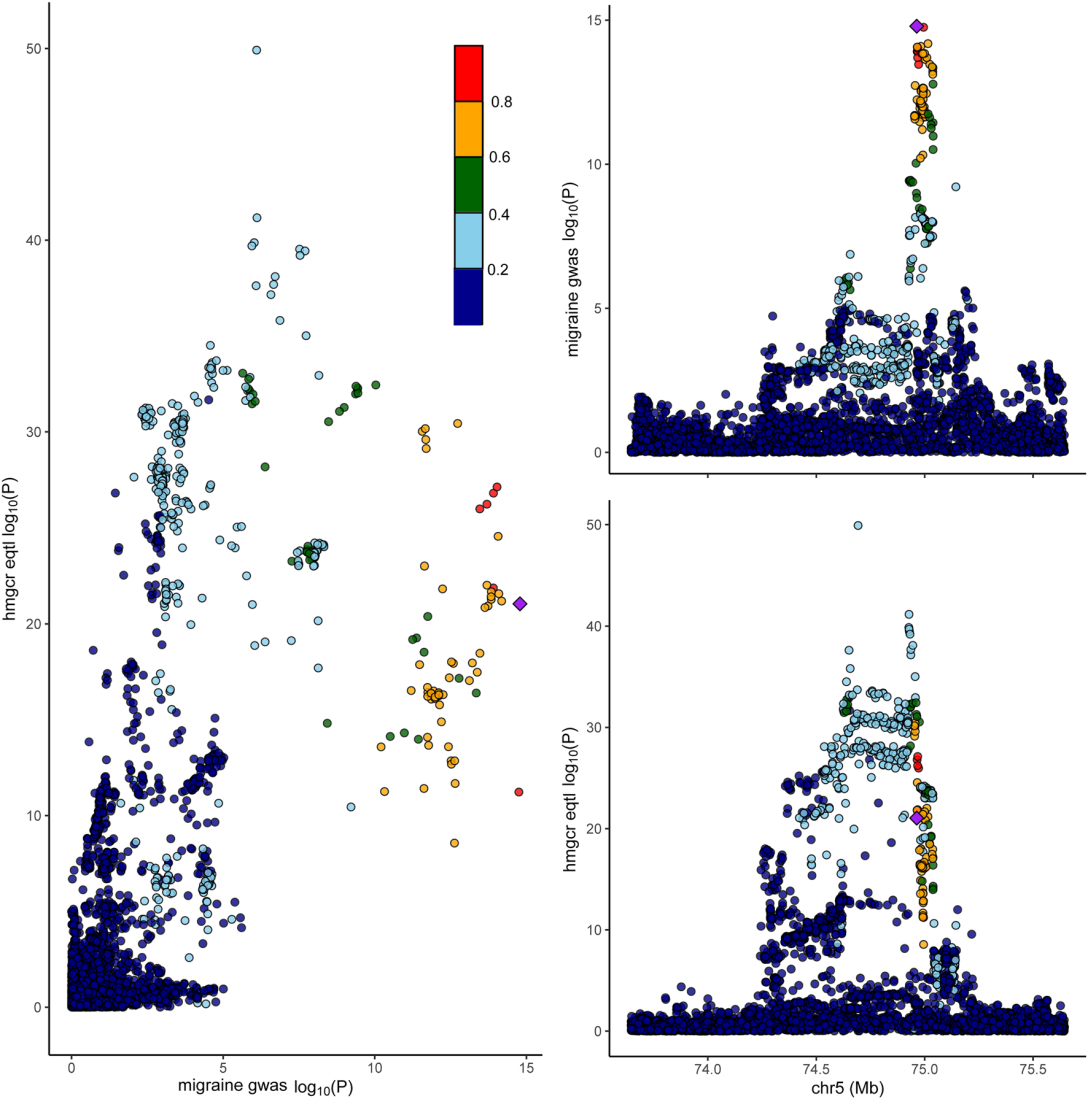
The visualization of HMG-CoA reductase gene expression and the colocalization analysis with migraine.

As shown in Fig. 2, the primary analysis of migraine data from the IHGC revealed that genetically predicted expression of *HMGCR* was associated with increased risk of migraine (OR = 1.55, 95% CI 1.30–1.84, *P* = 6.87 × 10^−7^). The replication study using data from FinnGen produced similar results (OR = 1.38, 95% CI 1.14–1.67, *P* = 7.38 × 10^−4^). Both sensitivity analyses yielded similar estimates, and in the same direction (Supplementary Table 6). This finding indicated that HMGCR inhibitors may reduce the risk of migraine susceptibility. No statistically significant heterogeneity or horizontal pleiotropy was observed (Supplementary Table 6). When causal variants were present, Bayesian colocalization analysis using data from the IHGC suggested that HMGCR and migraine shared the same variants (Coloc.abf-PPH4 = 0.97, Fig. 3) (Supplementary Table 7).

**Figure 4:**
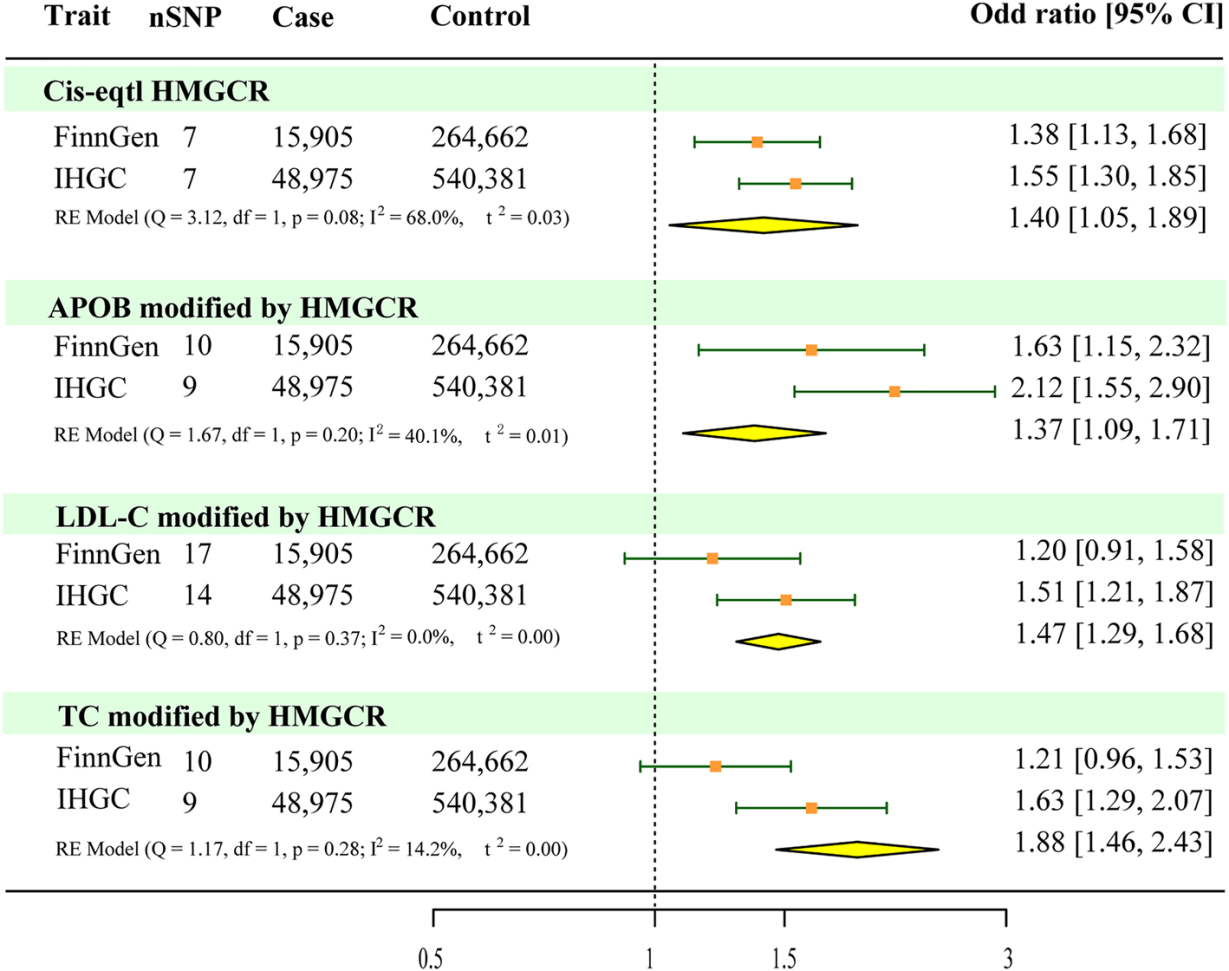
Meta-analysis of mendelian randomization analysis results based on inverse variance weighting.

The supplementary analyses suggested that genetically predicted levels of LDL (OR = 1.51, 95% CI 1.21–1.88, *P* = 2.50 × 10^−4^), TC (OR = 1.63, 95% CI 1.30–2.06, *P* = 2.93 × 10^−5^), and APOB (OR = 2.12, 95% CI 1.56–2.87, *P* = 1.35 × 10^−6^) modified by HMGCR were associated with an increased risk of migraine (Fig. 2, Supplementary Table 6). Replication analyses using data from the FinnGen study suggested that APOB (OR = 1.63, 95% CI 1.15–2.29, *P* = 5.60 × 10^−4^) modified by HMGCR were associated with an increased risk of migraine, but LDL levels (OR = 1.19, 95% CI 0.91–1.56, *P* = 0.20) and TC levels (OR = 1.21, 95% CI 0.95–1.53, *P* = 0.12) modified by HMGCR were not significantly associated with an increased risk of migraine (Fig. 2, Supplementary Table 6). No significant evidence of heterogeneity was observed using the IVW method (Supplementary Table 6). The intercept term of the MR–Egger regression and MR-PRESSO analyses indicated that horizontal pleiotropy was not significant (Supplementary Table 6). The results of the random-effects meta-analysis suggested that LDL, APOB, and TC levels were associated with an increased risk of migraine (Fig. 4). These findings supported the reliability of the MR studies. In summary, these results provide further support for the potential protective effects of HMGCR inhibitors against migraine.

**Figure 5:**
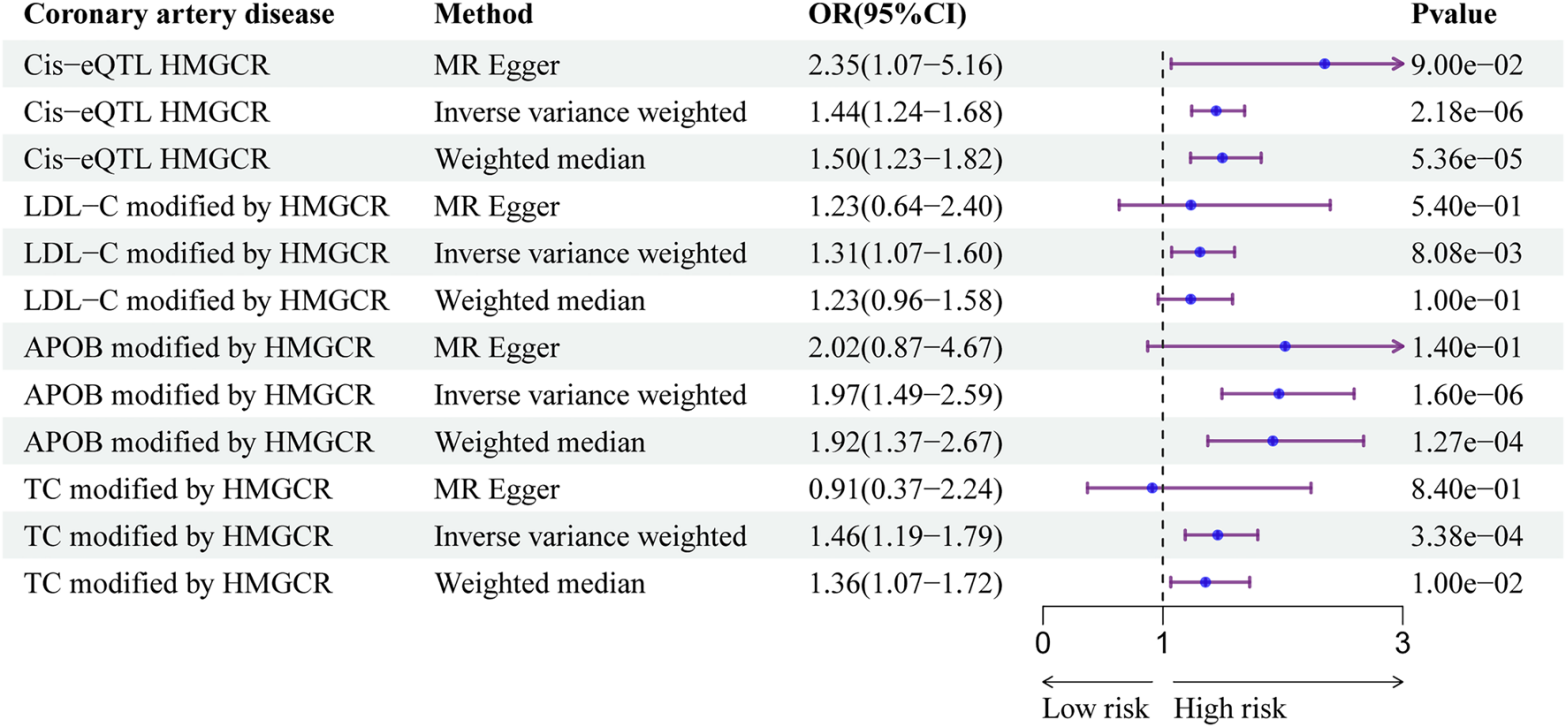
Associations between genetically proxied statins lipid-lowering drugs and the risk of coronary artery disease as a positive control analysis.

The genetically determined expression of HMGCR and the levels of LDC-C, APOB, and TC modified by HMGCR were associated with an increased risk of coronary artery disease (Fig. 5; Supplementary Table 8). As a reference for the positive control analyses, this result also increased the credibility of the included instrumental variables and confirmed the efficacy of the selected instruments.

## Discussion

Consistent results obtained from a rigorous MR analysis indicated that HMGCR expression and the circulating levels of three lipids (LDL-C, APOB, and TC) adjusted by HMGCR were significantly associated with an increased risk of migraine. These findings strongly suggest that HMGCR inhibitors hold promise as potential protective medications against migraines. In line with the prior study by Bi et al. it was found that HMGCR could potentially serve as a therapeutic target for migraines^45^. The merits of our study include: firstly, the inclusion of a greater number of cis-eQTLs that could influence HMGCR gene expression; secondly, the GWAS studies for migraines encompassed larger sample sizes and a greater number of migraine cases; thirdly, both the discovery and replication analyses indicated that a genetically determined high expression of HMGCR was correlated with an elevated risk of migraines. Nevertheless, this association did not seem to be directly linked to the total circulating levels of LDL-C, APOB, or TC, as we did not identify any significant correlation between these lipid levels and the risk of migraine.

In a recent meta-analysis examining the association between circulating lipids and migraine, individuals with migraine exhibited notably elevated levels of blood cholesterol, triglycerides, and LDL-C compared with healthy controls^46^. Discerning causality in observational research presents inherent difficulties, but the groundbreaking method of MR analysis has the potential to elucidate hypothetical causal relationships^17^. Unfortunately, a meticulous MR analysis here failed to reveal any substantial evidence for a causal relationship between the genetically determined levels of three circulating lipids and migraine.

Following the identification of a connection between circulating lipids and migraine in observational studies, researchers became intrigued by the potential therapeutic benefits of lipid-lowering medications in managing migraine. Statins are commonly used as prescription drugs in the field of neurology. Given the neurovascular nature of migraine, its etiology is influenced by inflammation and oxidative stress^47,48^, both regulated by statins^13,14^. The multifaceted properties of statins offer promising avenues for the advancement of migraine treatment. Furthermore, compelling evidence from animal studies suggests that statins may possess analgesic properties, further bolstering their potential as pain-relieving agents^49^. In a recent genetic association study conducted in a female migraine population, an intriguing link was discovered between migraines and specific lipoprotein subfractions, indicating a shared biological mechanism. The results of the colocalization analysis further identified this shared signal as circulating HMGCR, suggesting the potential effectiveness of statin analogs in the treatment of migraines^50^. Notably, exploring new applications of existing medications requires less time and resources than developing entirely new drugs de novo. In summary, statins have emerged as strong candidates for migraine treatment. Our MR study further supports this by highlighting the potential of HMGCR as a viable target for ameliorating migraine. Recent studies have focused primarily on the associations between statins, pain, and inflammation. For instance, in animal migraine models, atorvastatin suppressed B cell activation in the trigeminal caudal nucleus, demonstrating its ability to alleviate inflammation^49^. Similarly, in neuropathic pain models, pitavastatin demonstrated inhibitory effects on the JNK/P38/MAPK signaling pathway, resulting in a reduction in the release of inflammatory mediators^51^.

Nevertheless, existing research on the use of statins for migraine treatment presents conflicting results. While some case–control studies support the potential effectiveness of statins in alleviating migraine^52^, the results of several randomized controlled trials do not align with these results. These discrepancies could be attributed to several factors. First, the types of drugs used and their respective dosages were inconsistent across studies. Second, intervention measures within the control groups varied considerably. Third, the criteria for assessing migraine relief differed among studies. Finally, the durations of the interventions employed in the studies exhibited significant variation. Consequently, owing to the limited number of available studies, conducting a meta-analysis to quantitatively evaluate the efficacy of statins in the treatment of migraines would be premature. For further insight, please refer to Additional file 2, which provides a comprehensive overview of several published studies investigating the use of statins in migraine treatment. Based on current data, we find insufficient evidence to substantiate the use of statins as a definitive treatment for migraines. Further investigation is warranted to clarify the role of statins in migraine treatment across various layers of evidence, including genetic epidemiology.

In this MR investigation, we used genetic variants associated with HMGCR expression and HMGCR-mediated circulating lipid levels as instrumental variables for statins. Primary and supplemental analyses consistently supported HMGCR inhibitors as having the potential to reduce the risk of migraine. Hence, our study has significant implications, warranting case–control and randomized controlled studies to evaluate whether statins prevent migraine. Additionally, it supports a causal connection between HMGCR and migraines. Further investigations should include diverse populations and establish animal models specifically targeting migraine to comprehensively explore the effects and mechanisms of action of statins on migraine management.

Our study had several strengths. First, we employed genetic variants as instrumental variables instead of directly utilizing statins as the exposure, effectively minimizing confounding factors and mitigating the influence of unmeasured biases. Additionally, we incorporated four distinct types of instrumental variable and cross-validated our findings using data from two independent sources to enhance the reliability and robustness of our conclusions. Furthermore, to validate our instrumental variables, a positive control analysis was performed.

This study had some limitations. First, we were unable to identify suitable cis-acting pQTLs pertaining to HMGCR, which prevented us from establishing a clear association at the protein level between blood HMGCR levels and migraine. Second, despite the rigorous implementation of multiple sensitivity analyses to ensure that the MR assumptions were met, the presence of horizontal pleiotropy cannot be completely ruled out, which is an inherent limitation of MR studies. Third, the inclusion of populations of only European ancestry in the GWAS data restricts the generalizability of our findings. Therefore, caution should be exercised when extrapolating these associations to other populations. Fourth, we employed genetic variants in drug targets as proxies for the drug of interest rather than directly measuring drug-target inhibition, making direct comparisons challenging. Fifth, our analysis was limited to summary-level GWAS data, which restricted the depth of analysis of specific migraine subtypes. Finally, although the liver plays a crucial role in lipid metabolism, eQTLs data for this organ were not available.

## Conclusion

The findings of this careful MR study imply a causative link between HMGCR inhibition and migraine. These results warrant clinical studies to assess the efficacy of HMGCR inhibition as a therapeutic strategy. Further investigations should elucidate the underlying protective mechanisms associated with this inhibition.

### Supplementary Information

Supplementary Information 1.

Supplementary Information 2.

Supplementary Information 3.

## Data Availability

Genetic variants of 3 circulating lipids can be obtained through the original studies (https://doi.org/10.2337/db19-1134, https://doi.org/10.1371/journal.pmed.1003062, https://doi.org/10.1038/s41588-020-00757-z). Please visit the highly accessible eQTLGen consortium website at https://www.eqtlgen.org/ to get the GWAS summary data for cis-eQTLs. You can obtain the migraine GWAS summary data from the original study (https://doi.org/10.1038/s41588-021-00990-0) and from FinnGen (www.finngen.fi).

## Abbreviations

GWAS: Genome-wide association study
MR: Mendelian randomization
HMGCR: 3-Hydroxy-3-methylglutaryl coenzyme A reductase
LDL-C: Low-density lipoprotein cholesterol
APOB: Apolipoprotein B
TC: Total cholesterol
IHGC: International Headache Genetics Consortium
SNP: Single nucleotide polymorphism
eQTL: Expression quantitative trait locus
pQTL: Protein quantitative trait loci
cis-QTL: Cis-acting quantitative trait loci
LD: Linkage disequilibrium
IVW: Inverse variance weighting
MR-PRESSO: MR pleiotropy residual sum and outliers test
OR: Odds ratio
95% CI: 95% Confidence intervals
