# Supplementary material for: HMG-CoA reductase is a potential therapeutic target for migraine: a mendelian randomization study
Source: Sci Rep. 2024 May 27;14:12094. doi: 10.1038/s41598-024-61628-9 (PMC11130224; doi:10.1038/s41598-024-61628-9)
Supplement: Supplementary file 1 — Supplementary Information 1. [file 41598_2024_61628_MOESM1_ESM.docx]

**Role of statins in the prophylactic treatment of migraine: A literature review**

**Methods**

PubMed, Web of Science, Embase, and Cochrane Library databases were systematically searched. Keywords including migraine, statins, atorvastatin, and simvastatin were used to screen out relevant studies published before April 2023. We included prospective, interventional, controlled trials that evaluated the efficacy and/or safety of statin prophylaxis for migraine. Case reports, conference abstracts, trials with no reported results, animal studies, or studies not written in English were excluded. We extracted the following baseline data from the included studies: first author, publication year, study design, number of patients, age, detailed treatment exposures in the intervention and control groups, and study duration. Outcome indicators included frequency of headaches per month or number of headache attacks per month, proportion of patients with a 50% reduction in the frequency of migraine attacks, Visual Analogue Scale (VAS) score, and adverse drug effect occurrence.

**Literature evaluation**

**Atorvastatin use for the prophylactic treatment of migraine**

Sherafat et al. conducted a 24-week prospective triple-blind, RCT on 142 patients with a mean age of 29.6 ± 7.8 years, who were mostly women (67.6%).^1^ Patients were included if they were aged 18–65 years, had migraine with or without aura, experienced at least four episodes of migraine attack per month, and experienced at least two severe migraine attacks per month (which affected the QOL). Patients were excluded if they had mood disorder (detected using the Hamilton questionnaire), at least 15 migraine days per month, used atorvastatin for other disorders, used other therapeutic modalities during the last week (such as magnesium and herbal medicine), or had poor medication adherence. The enrolled patients were instructed not to take other medications during the study period. Sixty-eight patients were included in the final analysis, with 34 patients in the intervention group (atorvastatin 40 mg and nortriptyline 25 mg daily) and 34 patients in the control group (placebo and nortriptyline 25 mg daily). Outcome measures were evaluated based on data from periodic patient follow-up. At baseline, there was no significant between-group difference in the proportion of patients with >3 headache attacks per month (intervention vs. control: 61.8% vs. 82.6%, *P* = 0.20). At week 24, the intervention group had a significantly greater proportion of patients with <1 headache attack per month than the control group (85.5% vs. 47.0%, *P* = 0.004). However, there were no statistically significant between-group differences in the frequency distribution of migraine attacks at weeks 4 (*P* = 0.37) and 14 (*P* = 0.19). Overall, the odds ratio of developing a headache attack was reduced by 46% in the intervention group compared to the control group (OR = 0.54, *P* = 0.007). Therefore, the efficacy of atorvastatin use may be time-dependent. The QOL scores significantly increased in the intervention group from baseline compared with those in the control group at weeks 14 (17.71 ± 2.20 vs. 15.60 ±2.14, *P* = 0.001) and 24 (8.97 ± 1.35 vs. 17.47 ± 1.38, *P* = 0.001) of the study. Although the frequency of headache attacks decreased and QOL scores increased after treatment, there was no statistically significant between-group difference in headache VAS scores at weeks 4, 14, and 24 (*P* > 0.05 for each of the weeks). However, they observed a decrease in the mean VAS scores from baseline in both the intervention and control groups (6.0 ± 1.1 vs. 6.18 ± 1.2, respectively; *P* = 0.16). No patient withdrew from the study due to adverse drug reactions. The most common adverse reactions were myalgia, drowsiness, headache, dyspepsia, flatus, constipation, abdominal pain, skin rash, and weight gain; however, these clinical manifestations were usually alleviated during the course of the treatment, and no serious adverse reactions, such as rhabdomyolysis, myopathy and myalgia, were reported.

Ganji et al. conducted a 2-month prospective, randomized, triple-blind, placebo-controlled trial on 64 patients with a mean age of 36.8 ± 37.2 years, who were mostly women (67.2%).^2^ Patients were included if they were aged 18–65 years, had migraine with aura for at least 6 months, experienced at least three episodes of migraine attacks per month, and experienced fewer than three severe migraine attacks per month that negatively affect the QOL. Patients were excluded if they experienced more than 15 headache attacks per month, used statins for other diseases, or had significantly abnormal liver enzyme or creatine kinase levels. In addition, patients were instructed not to use other migraine medications (including nonsteroidal anti-inflammatory drugs) during the study. After 1 month of screening, the patients were randomly divided into intervention and control groups. Sixty-eight patients were recruited, 64 of whom were included in the final analysis. Patients in the intervention group (n = 33) were administered atorvastatin 20 mg and sodium valproate 500 mg daily, whereas those in the control group (n = 31) were administered placebo and sodium valproate 500 mg daily. The frequency of migraine attacks per month and the VAS scores of the participants were assessed using a questionnaire. At baseline, there were no differences in the frequency (4.67 ± 1.05 vs. 4.61 ± 1.09, *P* = 0.74) and severity (7.85 ± 1.03 vs. 7.90 ± 0.91, *P* = 0.89) of migraine attacks between the intervention and control groups. At the end of the study, the frequency of migraine attacks per month was significantly lower in the intervention group than in the control group (1.61 ± 0.75 vs. 3.61 ± 0.96, *P* = 0.0001). Moreover, the same finding was obtained after controlling for covariates. Hence, compared with the baseline value, the frequency of migraine attacks per month decreased by 3.06 and 1.0 in the intervention and control groups at the end of study, respectively. At month 2, the VAS score of migraine attacks was significantly lower in the intervention group than in the control group (3.27 ± 0.88 vs. 5.87 ± 1.03, *P* = 0.0001); the same result was obtained after controlling for covariates. After 2 months of treatment, patient satisfaction was significantly higher in the intervention group than in the control group (OR = 9.83, *P* = 0.001). No participant withdrew from the study because of adverse drug reactions. There was no significant between-group difference in the occurrence of adverse effects. There was no significant difference in the occurrence of adverse effects between the intervention and control groups (30.3% vs. 19.4%, respectively; *P* = 0.32). Common adverse reactions were gastrointestinal symptoms, joint or bone pain, myalgia, and skin rash; no serious adverse reactions were reported.

Hesami et al. conducted a 3-month randomized, double-blind clinical trial on 82 patients with a mean age of 33.42 ±9.09 years, who were mostly women (96.3%).^3^ Patients were included if they were aged 18–50 years, had migraine with aura for at least 6 months, and experienced 6 to 15 episodes of migraine attacks per month in the last 2 months. Patients were excluded if they were exposed to prophylactic treatment for migraine or had considerably abnormal liver enzyme levels. A total of 100 patients were recruited, 82 of whom were included in the final analysis. Patients in the intervention group (n = 46) were administered atorvastatin 40 mg daily, whereas those in the control group (n = 36) were administered sodium valproate 500 mg daily. On a daily basis, a neurologist assessed the frequency of headache attacks, number of analgesics used, and VAS scores of participants via phone calls. At baseline, there were no differences in the frequency (10.37 ± 3.25 vs. 11.14 ± 2.45, *P* = 0.18) and severity (7.91 ± 1.47 vs. 7.50 ± 1.80, *P* = 0.35) of migraine attacks between the intervention and control groups. At month 3, there was no significant between-group difference in the proportion of patients with more than 50% reduction in the number of headache attacks (intervention vs. control: 65.2% vs. 72.2%, *P* = 0.50). These findings were similar to those obtained at months 1 and 2. Furthermore, there was no significant between-group difference in the proportion of patients with more than 50% reduction in headache intensity at months 1, 2, and 3 of treatment; the highest proportions of patients with more than 50% reduction in headache intensity were observed at the end of study (intervention vs. control: 45.7% vs. 66.7%, *P* = 0.058). The proportion of patients with more than 50% reduction in headache attack duration peaked in the intervention and control groups at month 2 (73.9% vs. 75%, *P* = 0.91) and plateaued until the end of the study. There was no significant between-group difference in the number of patients whose mean monthly analgesic doses were decreased by more than 50% during the 3-month study (intervention vs. control: 50% vs. 52.8%, *P* = 0.803). One participant in each group withdrew from the study due to the following adverse drug reactions: drowsiness and menstrual disorder for the intervention and control groups, respectively. The number of reported cases of adverse reactions in the intervention group was less than that in the control group (32% vs. 66%, *P* = 0.002). The most common adverse reactions in the intervention group were dyspepsia, myalgia, weight gain, hair loss, dizziness. No serious adverse reactions occurred during the study period.

**Rrosuvastatin use for the prophylactic treatment of migraine**

Mazdeh et al. conducted a 4-week randomized, triple-blind, placebo-controlled trial on 120 patients with a mean age of 34.99 ± 10.41 years, who were mostly women (93.3%).^4^ Patients were included if they were aged ≥18 years, had a >3-year history of migraine, and experienced at least four episodes of migraine attacks per month. Patients with abnormal total cholesterol levels, hepatic or renal failure, and atherosclerotic disorders were excluded. Participants were instructed not to use other headache medications during the study period. All 120 recruited patients were included in the final analysis. Sixty patients were assigned to the intervention group and received rosuvastatin 10 mg daily and propranolol 10 mg twice daily; further, 60 patients were assigned to the control group and received propranolol 10 mg twice daily and placebo. Participants were followed up by professional staff via telephone questionnaires. At baseline, there was no difference in the frequency of migraine attacks per month between the intervention and control groups (16.20 ± 8.36 vs. 17.52 ± 9.56, *P* = 0.42). At week 4, there were significant between-group differences in the frequency of migraine attacks per month (intervention vs. control: 4.00 ± 7.20 vs. 10.12 ± 9.52, *P* < 0.001). Therefore, compared with the baseline values, the frequency of migraine attacks per month decreased by 12.2 and 7.4 in the intervention and control groups at the end of study, respectively. No statin-related side effects were reported.

**Simvastatin use for the prophylactic treatment of migraine**

Buettner et al. conducted a 24-week randomized, double-blind, placebo-controlled trial on 57 patients with a mean age of 33.89 ±14.93 years, who were mostly women (91.2%).^5^ Patients were included if they were aged ≥18 years, had a >3-year history of migraine, and experienced 3 to 14 episodes of migraine attacks per month. Patients with statin use for the treatment of other diseases, severe kidney disease, markedly abnormal creatine kinase and liver enzyme levels, or a history of long-term opioid use were excluded from the study. Participants were instructed to maintain a stable treatment regimen if they chose to continue using their own prophylactic medication during the study. After a 12-week washout period, 89 patients were randomized into two groups, 57 of whom were included in the final analysis. Patients in the intervention group (n = 28) were administered simvastatin 20 mg twice daily and vitamin D3 1000 units twice daily, whereas those in the control group (n = 29) were administered two matching placebos. The number of migraine attack days per month, number of analgesics used, and migraine disability assessment questionnaire (MIDAS) score of the participants were assessed through their headache diaries. At baseline, there was no difference in the number of migraine days per month (8.50 ± 4.81 vs. 6.00 ± 2.22, *P* = 0.05) and the number of days of migraine abortive medication use per month (6.83 ± 3.33 vs. 5.33 ± 2.47, *P* = 0.058) between the intervention and control groups. Compared with the baseline value, the change in the number of migraine attacks per month at 12 week was more pronounced in the intervention group than in the control group (-2.66 ± 2.96 vs. 0.33 ± 1.73, *P* < 0.001). Moreover, compared with the baseline value, the change in the number of days of migraine abortive medication use per month at 12 weeks was more pronounced in the intervention group than in the control group (-1.5 ± 2.46 vs. -0.33 ± 0.99, *P* = 0.002). These two findings remained unchanged at week 24. At week 12, the change in the MIDAS score compared with the baseline value was decreased more obviously in the intervention group than in the control group (-4.33 ± 5.35 vs. -1.33 ± 2.72, *P* = 0.002), albeit no between-group difference at week 24. At week 24, there was a significant between-group difference in the proportion of patients with more than 50% reduction in migraine days (intervention vs. control: 29% vs. 3%, *P* = 0.03). Common adverse reactions observed were myalgia, skin rash, and gastrointestinal symptoms. No serious adverse reactions occurred as a result of the drug treatment.

Medeiros et al. conducted a 90-day prospective, open-label study on 54 women. They included patients aged 18 to 45 years and who experienced more than six episodes of migraine attacks per month.^6^ In addition, patients in the intervention group, all of whom had hyperlipidemia, were administered simvastatin 20 mg daily, whereas those in the control group were administered propranolol 60 mg daily. All participants were instructed not to use other prophylactic medications during the study period. The frequency of headache attacks per month was assessed using participant headache diaries. In both groups, the frequency of migraine attacks per month was significantly lower at the end of the study than at baseline (*P* < 0.01 for both groups). In addition, there was no significant difference in the proportion of patients with more than 50% reduction in migraine attack frequency between the intervention and control groups (83% vs. 88%, *P* = 0.71). No serious side effects, such as rhabdomyolysis, myopathy and myalgia, occurred during this study. In the intervention group, three participants withdrew from the study early because of dizziness, nausea, abdominal pain, and insomnia; similarly, in the control group, three participants withdrew from the study because of symptomatic hypotension and malaise.

**Supplementary table 1. Basic characteristics of the included studies**

| Study | Study design | Sample  size | Age  (year) | Female  (%) | Intervention  group | Control  Group | Treatment duration | Primary outcomes | Secondary outcomes | Adverse drug reactions (%) |
| --- | --- | --- | --- | --- | --- | --- | --- | --- | --- | --- |
| Sherafat  2022 [23] | Triple-blind, randomized controlled trial | 68 | 29.6 ± 7.8 | 44 (64.7) | Atorvastatin 40 mg, nortriptyline 25 mg daily | Placebo, nortriptyline 25 mg daily | 24 weeks | Headache  frequency | Intensity, quality of life | 19 (27.9) |
| Ganji  2021 [24] | Triple-blind, randomized controlled trial | 64 | 36.8 ± 37.2 | 43 (67.2) | Atorvastatin 20 mg,  sodium valproate 500 mg daily | Sodium valproate 500 mg, placebo daily | 2 months | - | Intensity,  patient satisfaction | 16 (25.0) |
| Mazdeh  2020 [26] | Triple-blind, randomized controlled trial | 120 | 34.9 ±10.4 | 112 (93.3) | Propranolol 10 mg twice daily, rosuvastatin 10 mg daily | Propranolol 10 mg twice daily, placebo daily | 4 weeks | Migraine  frequency | - | 0 (0) |
| Hesami  2018 [25] | Randomized, double-blind, controlled trial | 82 | 33.4 ±9.1 | 79 (96.3) | Atorvastatin 40 mg daily | Sodium valproate 500 mg daily | 3 months | Headache  frequency | Intensity, duration of attacks, number of analgesics taken per attack | 39 (47.6) |
| Buettner  2015 [27] | Randomized, double-blind, placebo-controlled trial | 57 | 33.9 ±14.9 | 52 (91.2) | Simvastatin 20 mg twice daily, vitamin D3 1000 units twice daily | Placebo, placebo | 24 weeks | Headache  days | Number of analgesics taken per attack, migraine disability assessment questionnaire score | 9 (15.8) |
| Medeiros  2007 [28] | Prospective open-label | 54 | 18-45 | 54 (100) | Simvastatin 20 mg, daily | Propranolol 60 mg daily | 90 days | Headache  frequency | Headache  days | 6 (11.1) |
