# Supplementary material for: HMG-CoA reductase is a potential therapeutic target for migraine: a mendelian randomization study
Source: Sci Rep. 2024 May 27;14:12094. doi: 10.1038/s41598-024-61628-9 (PMC11130224; doi:10.1038/s41598-024-61628-9)
Supplement: Supplementary file 2 — Supplementary Information 2. [file 41598_2024_61628_MOESM2_ESM.docx]

**STROBE-MR checklist of recommended items to address in reports of Mendelian randomization studies**

| **Item No.** | **Section** | **Checklist item** | **Page No.** | **Relevant text from manuscript** |
| --- | --- | --- | --- | --- |
| 1 | **TITLE and ABSTRACT** | Indicate Mendelian randomization (MR) as the study’s design in the title and/or the abstract if that is a main purpose of the study | 1-2 | HMG-CoA reductase is a therapeutic target for migraine: a mendelian randomization study |
|  | **INTRODUCTION** |  |  |  |
| 2 | **Background** | Explain the scientific background and rationale for the reported study. What is the exposure? Is a potential causal relationship between exposure and outcome plausible? Justify why MR is a helpful method to address the study question | 2-4 | Studies have shown that certain lipid-lowering drugs such as statins possess migraine-ameliorating properties. Mendelian randomization (MR) provides a method that can be used to analyze causality. |
| 3 | **Objectives** | State specific objectives clearly, including pre-specified causal hypotheses (if any). State that MR is a method that, under specific assumptions, intends to estimate causal effects | 4 | In this study, we aimed to investigate the association between the risk of migraine and statin lipid-lowering agents using a two-sample drug-target MR design. To the best of our knowledge, this is the first MR study to study the association between migraine and the target of statin lipid-lowering drugs. |
|  | **METHODS** |  |  |  |
| 4 | **Study design and data sources** | Present key elements of the study design early in the article. Consider including a table listing sources of data for all phases of the study. For each data source contributing to the analysis, describe the following: | 5 | Given the observational association between circulating lipids and migraines, we first examined whether genetically predicted circulating lipids (LDL-C; apolipoprotein B, APOB; and total cholesterol, TC) were associated with migraines. Second, drug-targeted MR was performed to determine whether HMGCR expression in the blood affects migraines. Third, a colocalization analysis was performed to determine the presence of common genetic variants. Finally, to verify the observed associations, we assessed whether the levels of HMGCR-regulated LDL-C, APOB, and TC were associated with migraine. |
|  | a) | Setting: Describe the study design and the underlying population, if possible. Describe the setting, locations, and relevant dates, including periods of recruitment, exposure, follow-up, and data collection, when available. | 5-7 | Not applicable since this is a two-sample MR study based on summary-level data.  Summary-level GWAS data for circulating lipids were obtained from the UK Biobank. The circulating lipids included……… |
|  | b) | Participants: Give the eligibility criteria, and the sources and methods of selection of participants. Report the sample size, and whether any power or sample size calculations were carried out prior to the main analysis | 5-7 | Summary-level GWAS data for circulating lipids were obtained from the UK Biobank. The circulating lipids included…… |
|  | c) | Describe measurement, quality control and selection of genetic variants |  | Not applicable. |
|  | d) | For each exposure, outcome, and other relevant variables, describe methods of assessment and diagnostic criteria for diseases |  | Not applicable. |
|  | e) | Provide details of ethics committee approval and participant informed consent, if relevant | 5 | Informed consent and ethical approval had been obtained in all the original studies; therefore, no additional ethical approval was required for this study. |
| 5 | **Assumptions** | Explicitly state the three core IV assumptions for the main analysis (relevance, independence and exclusion restriction) as well assumptions for any additional or sensitivity analysis | 5 | The selection of valid instrumental variables must satisfy three assumptions of MR analysis |
| 6 | **Statistical methods: main analysis** | Describe statistical methods and statistics used |  |  |
|  | a) | Describe how quantitative variables were handled in the analyses (i.e., scale, units, model) | 5-7 | Summary-level GWAS data for circulating lipids……Triglycerides and high-density lipoprotein cholesterol were excluded because no instrumental variables that met the above criteria were extracted. |
|  | b) | Describe how genetic variants were handled in the analyses and, if applicable, how their weights were selected | 5-7 | genome-wide significance (p < 5 e-08) |
|  | c) | Describe the MR estimator (e.g. two-stage least squares, Wald ratio) and related statistics. Detail the included covariates and, in case of two-sample MR, whether the same covariate set was used for adjustment in the two samples | 5;7 | Age, sex, and type of genotyping chip were adjusted as covariates in the GWAS analyses of individuals of European ancestry included in the UK Biobank.  The cases included in this meta-analysis were adjusted for sex, age, and ancestry. |
|  | d) | Explain how missing data were addressed | 6 | Proxies not sought for instrumental variables not available in the outcomes |
|  | e) | If applicable, indicate how multiple testing was addressed | 8 | The Bonferroni method was employed to adjust the significance threshold for four exposures, requiring P < 1.25 × 10-2 |
| 7 | **Assessment of assumptions** | Describe any methods or prior knowledge used to assess the assumptions or justify their validity | 7-8 | The primary analytical method for MR is random-effects inverse variance-weighted (IVW), which assumes that all SNPs are valid instruments, allows for balanced pleiotropy, and provides the most precise estimates. |
| 8 | **Sensitivity analyses and additional analyses** | Describe any sensitivity analyses or additional analyses performed (e.g. comparison of effect estimates from different approaches, independent replication, bias analytic techniques, validation of instruments, simulations) | 7-8 | Additional sensitivity analyses included the MR–Egger intercept test, the weighted median test, the radial MR test, and the MR pleiotropy residual sum and outliers test. And visualization methods such as scatter plots and leave-one-out plots are also used to identify outliers. Heterogeneity among the different IVs was evaluated using Cochran's Q test. Burgess's online calculator was used to calculate the power of the MR estimates |
| 9 | **Software and pre-registration** |  |  |  |
|  | a) | Name statistical software and package(s), including version and settings used | 9 | R software (version 4.2.2; R Foundation for Statistical Computing, Vienna, Austria) was used for all statistical analyses . The R package for MR analysis included "TwoSampleMR (version 0.5.6)," "MR-PRESSO (version 1.0)," "RadialMR (version 1.0)," "Coloc (version 1.0)," and "Metafor (version 1.0) |
|  | b) | State whether the study protocol and details were pre-registered (as well as when and where) |  | Not applicable. |
|  | **RESULTS** |  |  |  |
| 10 | **Descriptive data** |  |  |  |
|  | a) | Report the numbers of individuals at each stage of included studies and reasons for exclusion. Consider use of a flow diagram |  | Not applicable. |
|  | b) | Report summary statistics for phenotypic exposure(s), outcome(s), and other relevant variables (e.g. means, SDs, proportions) | 5;7 | Summary-level GWAS data for circulating lipids were obtained from the UK Biobank. The circulating lipids included LDL-C (n = 431,167) (22), APOB (n = 439,214) (23), and TC (n = 342,508). Summary-level GWAS data for migraines were obtained from the International Headache Genetics Consortium (48,975 migraine cases and 540,381 controls) and FinnGen study (nCase = 1,5905, nControl = 264,662, R8 release) |
|  | c) | If the data sources include meta-analyses of previous studies, provide the assessments of heterogeneity across these studies |  | Not applicable. |
|  | d) | For two-sample MR:  i.  Provide justification of the similarity of the genetic variant-exposure associations between the exposure and outcome samples  ii.  Provide information on the number of individuals who overlap between the exposure and outcome studies | 7 | The majority of participants included in the included GWAS were of European ancestry.  There was no significant sample overlap between exposure and outcome. |
| 11 | **Main results** |  |  |  |
|  | a) | Report the associations between genetic variant and exposure, and between genetic variant and outcome, preferably on an interpretable scale |  | Not applicable. |
|  | b) | Report MR estimates of the relationship between exposure and outcome, and the measures of uncertainty from the MR analysis, on an interpretable scale, such as odds ratio or relative risk per SD difference |  | See supplementary table 4; supplementary table 6. |
|  | c) | If relevant, consider translating estimates of relative risk into absolute risk for a meaningful time period |  | Not applicable. |
|  | d) | Consider plots to visualize results (e.g. forest plot, scatterplot of associations between genetic variants and outcome versus between genetic variants and exposure) |  | Fig. 1; Fig 3. |
| 12 | **Assessment of assumptions** |  |  |  |
|  | a) | Report the assessment of the validity of the assumptions | 10 | The MR-Egger intercept did not report clear evidence for the presence of horizontal pleiotropy. The MR-PRESSO global test also did not report outliers. |
|  | b) | Report any additional statistics (e.g., assessments of heterogeneity across genetic variants, such as *I^2^*, Q statistic or E-value) | 10 | The heterogeneity test did not report significant heterogeneity. |
| 13 | **Sensitivity analyses and additional analyses** |  |  |  |
|  | a) | Report any sensitivity analyses to assess the robustness of the main results to violations of the assumptions | 10 | MR-Egger regression intercept analysis and MR-PRESSO analysis were used to assess the robustness of the results. |
|  | b) | Report results from other sensitivity analyses or additional analyses |  | supplementary table 6; Fig1-4 |
|  | c) | Report any assessment of direction of causal relationship (e.g., bidirectional MR) |  | Not applicable. |
|  | d) | When relevant, report and compare with estimates from non-MR analyses |  | Not applicable. |
|  | e) | Consider additional plots to visualize results (e.g., leave-one-out analyses) |  | Not applicable |
|  | **DISCUSSION** |  |  |  |
| 14 | **Key results** | Summarize key results with reference to study objectives | 11 | Consistent results obtained from a rigorous MR analysis indicated that HMGCR expression and the circulating levels of three lipids (LDL-C, APOB, and TC) adjusted by HMGCR were significantly associated with an increased risk of migraine. |
| 15 | **Limitations** | Discuss limitations of the study, taking into account the validity of the IV assumptions, other sources of potential bias, and imprecision. Discuss both direction and magnitude of any potential bias and any efforts to address them | 13-14 | Nevertheless, existing research on the use of statins for migraine treatment presents conflicting results……role of statins in migraine treatment across various layers of evidence, including genetic epidemiology. |
| 16 | **Interpretation** |  |  |  |
|  | a) | Meaning: Give a cautious overall interpretation of results in the context of their limitations and in comparison with other studies | 15 | The findings of this careful MR study imply a causative link between HMGCR inhibition and migraine. |
|  | b) | Mechanism: Discuss underlying biological mechanisms that could drive a potential causal relationship between the investigated exposure and the outcome, and whether the gene-environment equivalence assumption is reasonable. Use causal language carefully, clarifying that IV estimates may provide causal effects only under certain assumptions | 12 | Given the neurovascular nature of migraine, its etiology is influenced by inflammation and oxidative stress, both regulated by statins . The multifaceted properties of statins offer promising avenues for the advancement of migraine treatment. Furthermore, compelling evidence from animal studies suggests that statins may possess analgesic properties, further bolstering their potential as pain-relieving agents |
|  | c) | Clinical relevance: Discuss whether the results have clinical or public policy relevance, and to what extent they inform effect sizes of possible interventions | 15 | These results warrant clinical studies to assess the efficacy of HMGCR inhibition as a therapeutic strategy. Further investigations should elucidate the underlying protective mechanisms associated with this inhibition. |
| 17 | **Generalizability** | Discuss the generalizability of the study results (a) to other populations, (b) across other exposure periods/timings, and (c) across other levels of exposure | 13 | This MR study was based on data drawn from subjects of predominantly European ancestry, thus limiting the extrapolation of our findings to other ethnic populations. |
|  | **OTHER INFORMATION** |  |  |  |
| 18 | **Funding** | Describe sources of funding and the role of funders in the present study and, if applicable, sources of funding for the databases and original study or studies on which the present study is based | 17 | This work was supported by a grant from the Natural Science Foundation of Jilin Province of China (Grant no. 20200201606JC), Scientific Research Program of Jilin Health and Family Planning Commission (Grant No. 2016J049) and the National Natural Science Foundation of China (Grant No. 31872772) to Ming Dong. |
| 19 | **Data and data sharing** | Provide the data used to perform all analyses or report where and how the data can be accessed, and reference these sources in the article. Provide the statistical code needed to reproduce the results in the article, or report whether the code is publicly accessible and if so, where | 18 | Genetic variants of 3 circulating lipids can be obtained through the original studies (https://doi.org/10.2337/db19-1134, https://doi.org/10.1371/journal.pmed.1003062, https://doi.org/10.1038/s41588-020-00757-z). Please visit the highly accessible eQTLGen consortium website at https://www.eqtlgen.org/ to get the GWAS summary data for cis-eQTLs. You can obtain the migraine GWAS summary data from the original study (https://doi.org/10.1038/s41588-021-00990-0) and from FinnGen (www.finngen.fi). |
| 20 | **Conflicts of Interest** | All authors should declare all potential conflicts of interest | 17 | The authors declare that they have no conflicts of interest. |

This checklist is copyrighted by the Equator Network under the Creative Commons Attribution 3.0 Unported (CC BY 3.0) license.

1. Skrivankova VW, Richmond RC, Woolf BAR, Yarmolinsky J, Davies NM, Swanson SA, et al. Strengthening the Reporting of Observational Studies in Epidemiology using Mendelian Randomization (STROBE-MR) Statement. JAMA. 2021;under review.

2. Skrivankova VW, Richmond RC, Woolf BAR, Davies NM, Swanson SA, VanderWeele TJ, et al. Strengthening the Reporting of Observational Studies in Epidemiology using Mendelian Randomisation (STROBE-MR): Explanation and Elaboration. BMJ. 2021;375:n2233.
